# Spatial metabolomics identifies distinct tumor-specific and stroma-specific subtypes in patients with lung squamous cell carcinoma

**DOI:** 10.1038/s41698-023-00434-4

**Published:** 2023-11-02

**Authors:** Jun Wang, Na Sun, Thomas Kunzke, Jian Shen, Philipp Zens, Verena M. Prade, Annette Feuchtinger, Sabina Berezowska, Axel Walch

**Affiliations:** 1 Research Unit Analytical Pathology, Helmholtz Zentrum München–German Research Center for Environmental Health, Neuherberg, 85764 Germany; 2 Institute of Tissue Medicine and Pathology, University of Bern, Murtenstrasse 31, 3008 Bern, Switzerland; 3 Graduate School for Health Sciences, University of Bern, Mittelstrasse 43, Bern, 3012 Switzerland; 4 Department of Laboratory Medicine and Pathology, Institute of Pathology, Lausanne University Hospital and University of Lausanne, Lausanne, 1011 Switzerland

**Keywords:** Cancer metabolism, Non-small-cell lung cancer

## Abstract

Molecular subtyping of lung squamous cell carcinoma (LUSC) has been performed at the genomic, transcriptomic, and proteomic level. However, LUSC stratification based on tissue metabolomics is still lacking. Combining high-mass-resolution imaging mass spectrometry with consensus clustering, four tumor- and four stroma-specific subtypes with distinct metabolite patterns were identified in 330 LUSC patients. The first tumor subtype T1 negatively correlated with DNA damage and immunological features including CD3, CD8, and PD-L1. The same features positively correlated with the tumor subtype T2. Tumor subtype T4 was associated with high PD-L1 expression. Compared with the status of subtypes T1 and T4, patients with subtype T3 had improved prognosis, and T3 was an independent prognostic factor with regard to UICC stage. Similarly, stroma subtypes were linked to distinct immunological features and metabolic pathways. Stroma subtype S4 had a better prognosis than S2. Subsequently, analyses based on an independent LUSC cohort treated by neoadjuvant therapy revealed that the S2 stroma subtype was associated with chemotherapy resistance. Clinically relevant patient subtypes as determined by tissue-based spatial metabolomics are a valuable addition to existing molecular classification systems. Metabolic differences among the subtypes and their associations with immunological features may contribute to the improvement of personalized therapy.

## Introduction

Lung squamous cell carcinoma (LUSC) and lung adenocarcinoma (LUAD) are the most common histological subtypes of non-small cell lung cancer (NSCLC), which accounts for almost 85% of all human lung cancers^1^. Unlike LUAD, patients with LUSC have not benefited from targeted therapies^2–4^, and there are substantial differences of LUSC treatment responses among current therapeutic options^5^. There continues to be great interest in investigating additional predictive biomarkers to facilitate the selection of those patients with LUSC who are most likely to benefit from chemotherapy, immunotherapy, targeted therapy, and other novel agents. To address this issue, research is now focusing on the development of classification systems based on multiple molecular levels, including genomics, transcriptomics, and proteomics, which would aid in understanding LUSC and in subsequently identifying therapeutic vulnerabilities and achieving effective, biomarker-based patient stratification.

Genomic and transcriptomic technologies have provided important insights into the molecular underpinnings of LUSC, leading to preliminary patient stratification strategies^6–10^. The Cancer Genome Atlas established four LUSC-related gene subtypes associated with cell cycle and apoptosis, antioxidant gene expression, phosphatidylinositide 3-kinase signaling, and epigenetic signaling^6^. In addition, two recent studies performed comprehensive proteogenomic characterization of LUSC^11,12^. One of these identified five distinct molecular subtypes by multiomic clustering analysis: the basal-inclusive subtype, classical subtype, EMT-enriched subtype, inflamed-secretory subtype, and proliferative-primitive subtype^12^. Based on these molecular classification results, research such as the NCI’s Molecular Analysis for Therapy Choice trial is attempting to capitalize on improved molecular knowledge of LUSC to employ precision therapy^13^.

Combining multiple immunological markers, such as programmed cell death protein 1 (PD1), programmed death ligand 1 (PD-L1), cluster of differentiation 3 (CD3), and cluster of differentiation 8 (CD8), with established molecular subtypes may increase the predictive robustness and guide the implementation of NSCLC precision medicine^7^. The genomic and transcriptomic alterations in LUSC shape the functional proteome, control the infiltration of immune cells, and present potential vulnerabilities that can be exploited therapeutically. The immune checkpoint pathway has been shown to play a crucial role in mediating immune tolerance in NSCLC, with antibody agents that block this pathway (e.g., agents against PD1/PD-L1) producing durable responses^14,15^, and where expression of checkpoint markers correlates with treatment efficacy^5^. Alternative markers for checkpoint blockade response, including T-cell immunohistochemistry and other immunological markers, are also being considered^16–18^.

Many important clinical advances in LUSC have been driven by genomic and proteomic profiling of bulk tumor material, and thus we anticipate that the same will prove true of bulk metabolomic characterization in the LUSC tissues. Recently, one study demonstrates the feasibility of an ensemble machine learning approach to accurately predict NSCLC patient survival from tumor core biopsy metabolomic data^19^, while another study suggests that metabolomic analysis of lung tumor core biopsies can differentiate patients into low- and high-risk groups based on survival events and probability^20^. The two studies applied liquid chromatography-tandem mass spectrometry (LC-MS/MS) and showed great promise of metabolomics in identifying diagnostic and prognostic biomarkers for NSCLC patients in clinical practice^19,20^. High-mass-resolution matrix-assisted laser desorption-ionization (MALDI) imaging mass spectrometry (IMS) directly enables the detection and localization of thousands of different molecules within a routinely preserved tissue section, allowing for the discrimination of tumor and stroma regions in NSCLC tissues^21^ and tumor subtyping^22–24^. The metabolic compositions of both tumor and stroma regions were able to provide rich molecular information and may contribute to estimating prognosis in patients diagnosed with NSCLC. Spatial metabolomics enables immunophenotype-guided in situ metabolomics, facilitating the automated and objective identification of histological and functional features in intact tissue sections and the comprehensive analyses of metabolic constitutions of tumor and the stroma regions from large-scale clinical cohort studies^25^.

This is the first large-scale study to stratify LUSC patients based on their tissue metabolic profiles. High-mass-resolution MALDI-IMS combined with consensus clustering analysis was applied to establish metabolic classification based on tumor- and stroma-specific tissue regions in LUSC patients. The results were tested in an independent cohort to demonstrate the ability of metabolic subtypes to associate with the response to chemotherapy. The metabolic constitution in LUSC provides an alternative option with which to stratify LUSC patients.

## Results

### Identification of LUSC patient subtype based on metabolite profiling

**Fig. 1:**
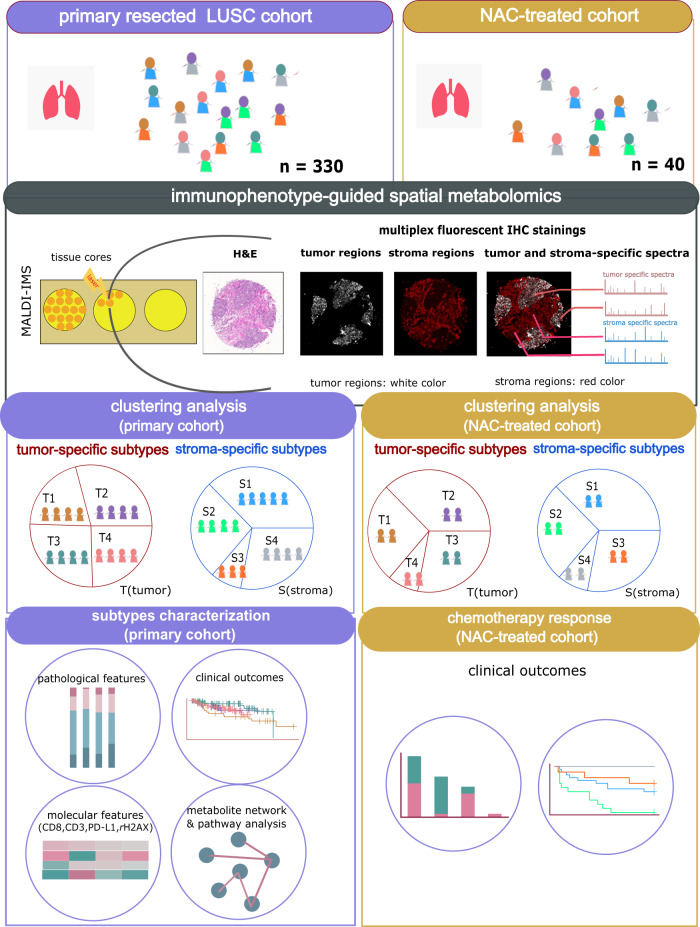
Study design for combining spatial metabolomics with consensus clustering analysis to stratify LUSC patients. LUSC patients were analyzed with spatial metabolomics by MALDI–IMS. The pipeline includes immunophenotype-guided spatial metabolomics, data preprocessing, and data analysis. Separate consensus clustering analyses were performed using the metabolic features evaluated in tumors and the stroma, resulting in tumor and stroma-specific subtypes. The tumor and stroma-specific subtypes of the primary resected cohort were then characterized by clinicopathological features, clinical outcomes, molecular features (immunological features and DNA damage marker), and specific metabolic pathways. The independent NAC-treated cohort was applied to associate chemotherapy responses with the established metabolic subtypes.

**Fig. 2:**
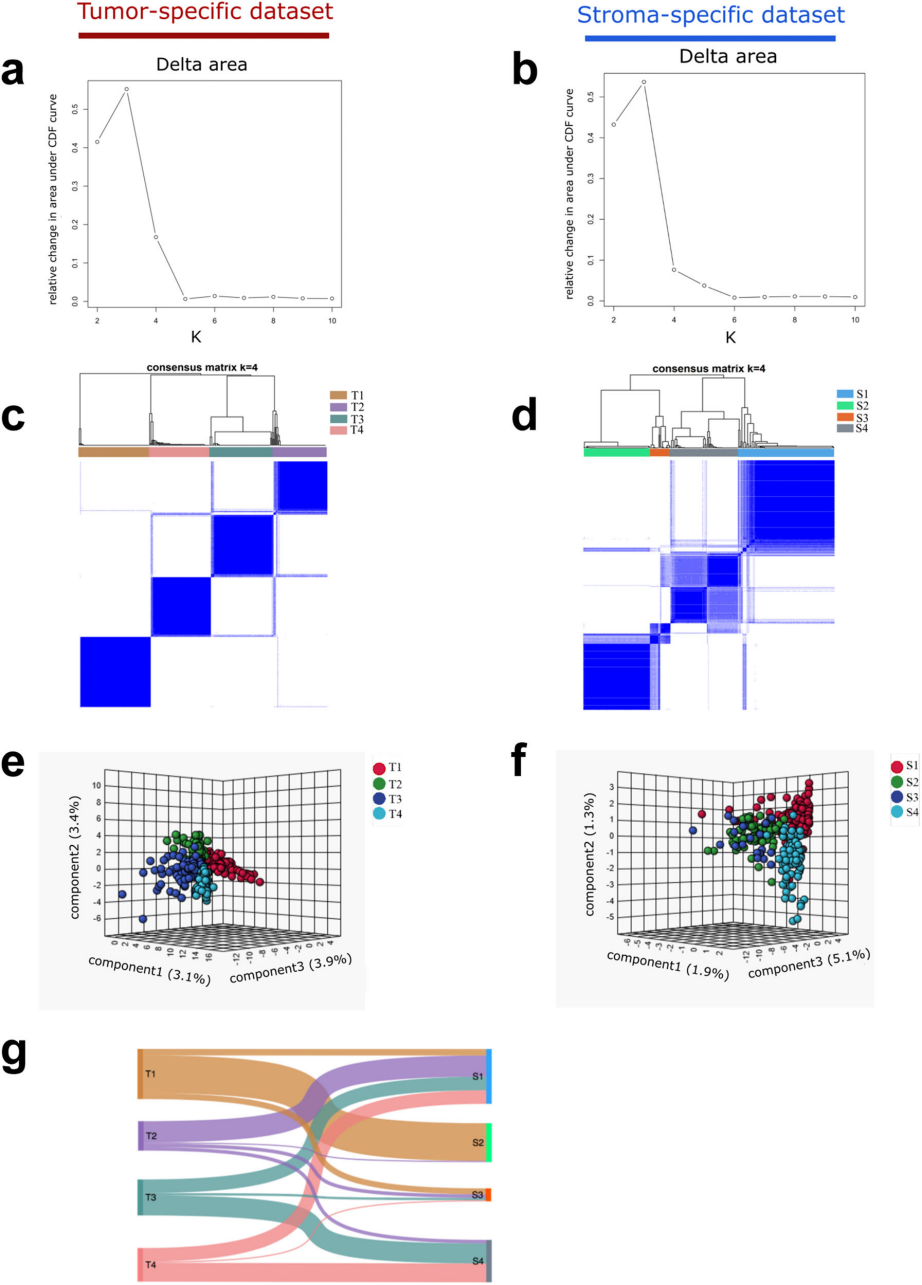
Identification of tumor- and stroma-specific subtypes and their association with molecular features. The relative change in the area under CDF curve of **a** tumor and **b** stroma datasets. The number of clusters is changed from 2 to 10. Delta area plot reflecting the relative changes in the area under the CDF curve. Setting the number of clusters to 4 leads to the relative increase in area became noticeably smaller; this number was thus selected as the optimal number of clusters. Consensus matrix heatmap of the chosen four clusters of **c** tumor- and **d** stroma-specific datasets. A color gradient ranging between 0 and 1 is defined as the average consensus value for all pairs of individuals. A value closer to 1 indicates better cluster stability. Three-dimensional sPLSDA analysis suggests that patients could be stratified into four subtypes in both **e** tumor- and **f** stroma-specific datasets. Points representing samples are colored according to the metabolic subtypes of patients. **g** Alluvial diagram depicts the relationship of tumor- and stroma-specific subtypes. CDF cumulative distribution function, sPLSDA sparse partial least-squares discriminant analysis.

A schematic overview of the conceptual methodology in this study is shown in Fig. 1. To determine whether tumor and stroma regions in the primary resected patient cohort had significant differences in metabolite composition, we performed tumor and stroma region-specific unsupervised consensus clustering analysis. Consensus matrix heatmaps and cumulative distribution function (CDF) plots were drawn to determine the optimal number of clusters. The delta area plot shown in Fig. 2a, b reflects the relative changes in the area under the CDF curve. The largest changes in area for tumor-specific and stroma-specific data occurred when the number of clusters was set to 4, at which point the relative increase in area became noticeably smaller. Thus, the optimal cluster numbers for both tumor-specific and stroma-specific data were set to 4. Color-coded consensus heatmaps were obtained by applying consensus clustering to tumor- and stroma-specific datasets (Fig. 2c, d). As shown in Fig. 2c, the blocks are barely overlap in the heatmap, indicating that the four clusters could be distinguished on tumor-specific spectra. The four stroma-specific clusters also have clean boundaries, indicating good cluster stability over repeated iteration (Fig. 2d). Of the 313 tumor regions, 91 were assigned to subtype T1 (29%), 64 to T2 (20%), 81 to T3 (26%), and 77 to T4 (25%). Furthermore, of the 268 stroma regions, 100 were assigned to subtype S1 (37%), 71 to subtype S2 (27%), 22 to subtype S3 (8%), and 75 to subtype S4 (28%).

To estimate the ability of MALDI-IMS data to distinguish LUSC subtypes, we additionally assessed the variance among molecular subtypes using sparse partial least-squares discriminant analysis (sPLSDA). The results revealed clear separation of both tumor- and stroma-specific subtypes, indicating that they could be readily distinguished based on metabolite levels (Fig. 2e, f). The alluvial diagram shown in Fig. 2g indicates the distribution of patients between tumor- and stroma-specific subtypes. Subtype similarities are observed between T1 and S2.

### Correlation of tumor- and stroma-specific subtypes with immunological features and DNA damage

**Fig. 3:**
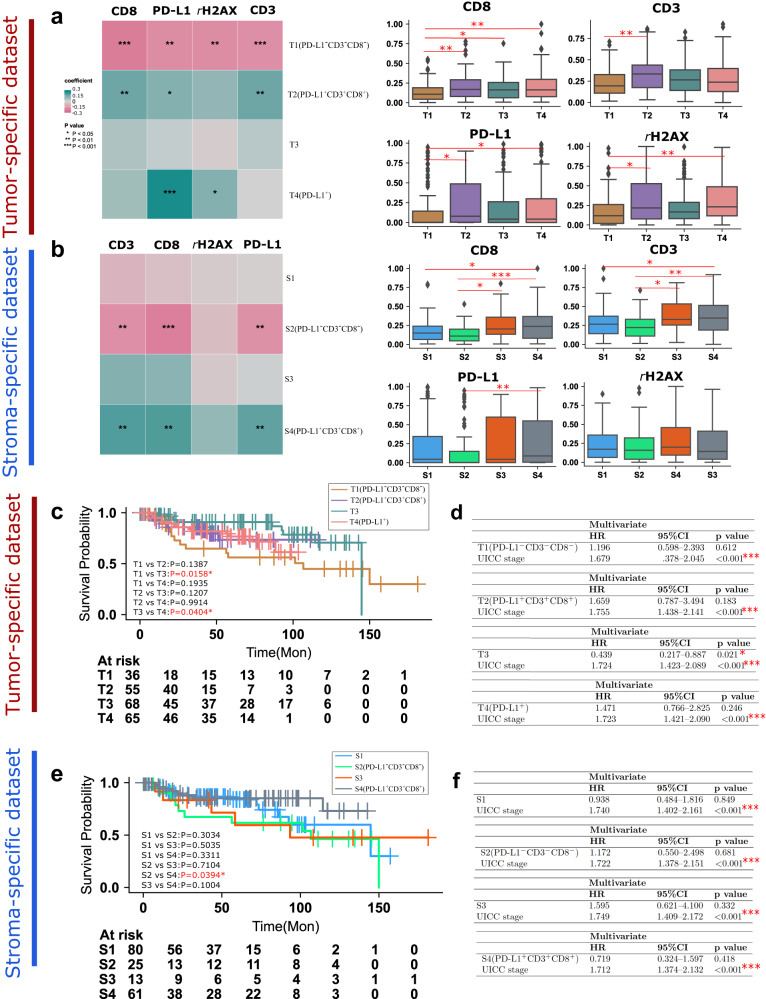
Association of metabolic subtypes with molecular features and prognosis. Molecular features (CD8, CD3, PD-L1, and *γ*H2AX) significantly associated with tumor- (**a**, left) and stroma-specific (**b**, left) subtypes by Spearman’s rank-order correlation analysis and the distribution of expression per molecular feature in each subtype as shown by boxplots (**a** and **b**, right). Each box plot displays the interquartile range (IQR), with the lower boundary representing the 25th percentile and the upper boundary representing the 75th percentile. The line within the box displays the median, and the whiskers extend to ±1.5 × IQR. Two-sided *p* value was calculated by Kruskal–Wallis test and post hoc Dunn’s multiple comparison test. **c** Survival analysis of tumor-specific subtypes using Kaplan–Meier curves by log-rank test and **d** multivariate Cox proportional hazard analysis of tumor-specific subtypes as well as UICC stage. T3 remains significant in multivariate analysis, indicating that it is a factor independently predictive of patient survival. **e** Survival analysis of stroma-specific subtypes using Kaplan–Meier curves by log-rank test and **f** multivariate Cox proportional hazard analysis of stroma-specific subtypes as well as UICC stage. * represents two-sided *p* < 0.05, ** represents two-sided *p* < 0.01, *** represents two-sided *p* < 0.001.

To explore differences in tumor- and stroma-specific subtypes, we investigated their associations with DNA damage (*γ*H2AX expression) and immunological features including cluster of differentiation 3 (CD3), cluster of differentiation 8 (CD8), and programmed death ligand 1 (PD-L1). All associations of those features with tumor-specific subtypes and stroma-specific subtypes are shown in Fig. 3a, b (left) and Supplementary Tables 2 and 3. Among the four tumor-specific subtypes (Fig. 3a), PD-L1 (*p* = 0.0012), CD3 (*p* = 0.0002), CD8 (*p* = 0.0001), and *γ*H2AX (*p* = 0.0016) are positively correlated with tumor-specific subtype T1. Conversely, tumor-specific subtype T2 is negatively correlated with PD-L1 (*p* = 0.0390), CD3 (*p* = 0.0071), CD8 (*p* = 0.0080), and *γ*H2AX (*p* = 0.0933). No significant correlation is found between T3 and these features. Tumor-specific subtype T4 is positively correlated with PD-L1 (*p* = 0.0004) and *γ*H2AX (*p* = 0.0333). Meanwhile, T4 shows no significant correlation with CD3 (*p* = 0.9919) or CD8 (*p* = 0.1755). Based on these results, we categorize the tumor-specific subtype with negative associations with PD-L1, CD3, and CD8 as T1(PD-L1^-^CD3^-^CD8^-^), that with positive associations with PD-L1, CD3, and CD8 as T2(PD-L1^+^CD3^+^CD8^+^), that with elevated PD-L1 protein expression as T4(PD-L1^+^), and the remaining tumor subtype as T3. The distribution of the expression of all immunological features and DNA damage in the tumor-specific subtypes is shown as boxplots in Fig. 3a (right).

As shown in Fig. 3b, stroma-specific subtype S2 is negatively associated with PD-L1 (*p* = 0.0058), CD3 (*p* = 0.0041), CD8 (*p* = 0.3660), and *γ*H2AX (*p* = 0.1242). In contrast, stroma-specific subtype S4 is positively associated with PD-L1 (*p* = 0.0056), CD3 (*p* = 0.0019), CD8 (*p* = 0.0017), and *γ*H2AX (*p* = 0.1242). No significant correlations with these features are found in S1 and S3. Thus, stroma-specific subtypes are accordingly renamed S1, S2(PD-L1^-^CD3^-^CD8^-^), S3, and S4(PD-L1^+^CD3^+^CD8^+^). The distribution of the expression of all immunological features and DNA damage in stroma-specific subtypes is shown as boxplots in Fig. 3b (right).

### Association of tumor-specific and stroma-specific subtypes with patient prognosis and clinicopathological features

The potential differences in prognosis among the tumor- and stroma-specific subtypes were analyzed. The Kaplan–Meier curve and log-rank test indicate better outcomes for subtype T3 than for T1(PD-L1^-^CD3^-^CD8^-^) (*p* = 0.0158) and T4 (PD-L1^+^) (*p* = 0.0404) (Fig. 3c). No statistically significant differences are observed in other pairwise tumor-specific subtype comparisons or overall in the four tumor-specific subtype comparisons. The multivariate Cox regression analysis shows that T3 can serve as a subtype with an independent effect on prognosis with regard to the UICC classification system (*p* = 0.021, HR = 0.439) (Fig. 3d). In the stroma-specific subtypes, S4(PD-L1^+^CD3^+^CD8^+^) has a better prognosis than S2(PD-L1^-^CD3^-^CD8^-^) (*p* = 0.0394). Survival does not differ significantly in other pairwise subtype comparisons or in an overall comparison of the four subtypes (Fig. 3e). None of the stroma-specific subtypes is found to serve as independent predictors of prognosis with regard to the UICC classification system (Fig. 3f).

We next investigated whether tumor-specific and stroma-specific subtypes differed in the most common clinicopathological characteristics. In all tumor-specific and stroma-specific subtypes, no associations were found with age, sex, resection status, grade, UICC stage, or TNM stage (Supplementary Fig. 1).

### Tumor- and stroma-specific metabolic subtypes with distinct metabolites and related metabolic pathways

**Fig. 4:**
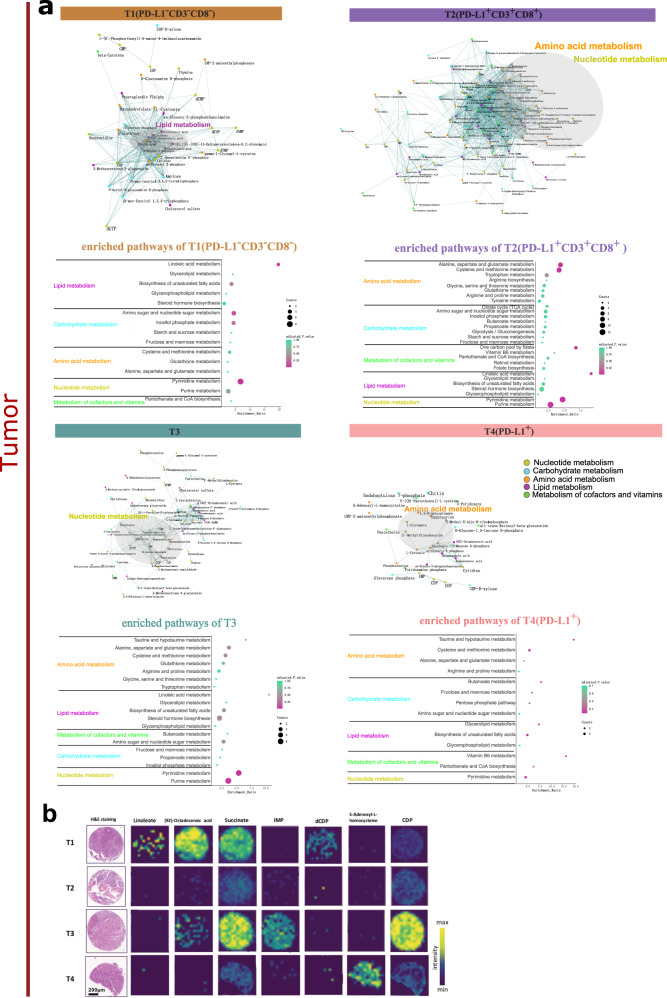
Metabolite characteristics and enriched pathways of tumor subtypes. **a** (top) Correlation networks of endogenous metabolites within each of the four tumor-specific subtypes. Correlations between metabolites were calculated and filtered (adjusted two-sided *p* < 0.001). Edges represent positive (green) and negative (pink) correlations between metabolites. Node color in the network indicates metabolic pathways. **a** (bottom) Quantitative enrichment pathway analysis within each of the four tumor-specific subtypes. Pathways enriched in each of the tumor-specific subtypes are represented by scatter plots. The x-axis indicates the pathway enrichment ratio, and the y-axis indicates the pathway term. Dot color indicates the adjusted *p* value. Dot size indicates the counts of metabolites. **b** Ion distribution maps of representative metabolites in the tumor-specific subtypes. Linoleate and (9Z)-Octadecenoic acid are selected from the correlation network T1(PD-L1^-^CD3^-^CD8^-^). dCDP is selected from the correlation network T1(PD-L1^-^CD3^-^CD8^-^) and T2(PD-L1^+^CD3^+^CD8^+^). Succinate shows in the correlation networks T1(PD-L1^-^CD3^-^CD8^-^) and T3. CDP and IMP are selected from the correlation network T3. S-Adenosyl-L-homocysteine is selected from the correlation network T4(PD-L1^+^). IMP inosine monophosphate, CDP cytidine diphosphate, dCDP 2’-deoxycytidine diphosphate.

To obtain a deeper insight into the underlying differences in metabolites among the metabolic subtypes, a correlation network analysis and quantitative enrichment analysis were conducted based on each of the tumor- and stroma-specific subtypes, and significantly correlated metabolites of each subtype were identified and visualized as networks and pathways as shown in Fig. 4a and Supplementary Fig. 2. As shown in Fig. 4a, the dense cluster and enriched pathways in T1(PD-L1^-^CD3^-^CD8^-^) indicates a correlation of lipid metabolism and pyrimidine metabolism. For the T2(PD-L1^+^CD3^+^CD8^+^) subtype, there are correlations of metabolites involved in amino acid metabolism and nucleotide metabolism. For the T3 subtype, there are multiple correlations of metabolites involved in nucleotide metabolism. T4(PD-L1^+^) is representatively characterized by amino acid metabolism. As shown in Supplementary Fig. 2, the most representative pathways are amino acid metabolism and nucleotide metabolism in S1 subtype. Similar to the T1(PD-L1^-^CD3^-^CD8^-^) subtype, S2(PD-L1^-^CD3^-^CD8^-^) demonstrates a correlation of lipid metabolism and pyrimidine metabolism. S3 is representatively characterized by lipid metabolism. For the S4 subtype, there are correlations of metabolites involved in nucleotide metabolism. Figure 4b showed the spatial distribution of representative metabolites selected from correlated networks of tumor-specific subtypes. The above results demonstrate that tumor- and stroma-specific subtypes are correlated with diverse metabolites and metabolic pathways. Specifically, the subtype similarities of enriched metabolic pathways are observed between T1(PD-L1^-^CD3^-^CD8^-^) and S2(PD-L1^-^CD3^-^CD8^-^).

### Three stroma-specific subtypes correlate with chemotherapy efficiency in an independent neoadjuvant chemotherapy-treated cohort (NAC-treated cohort)

**Fig. 5:**
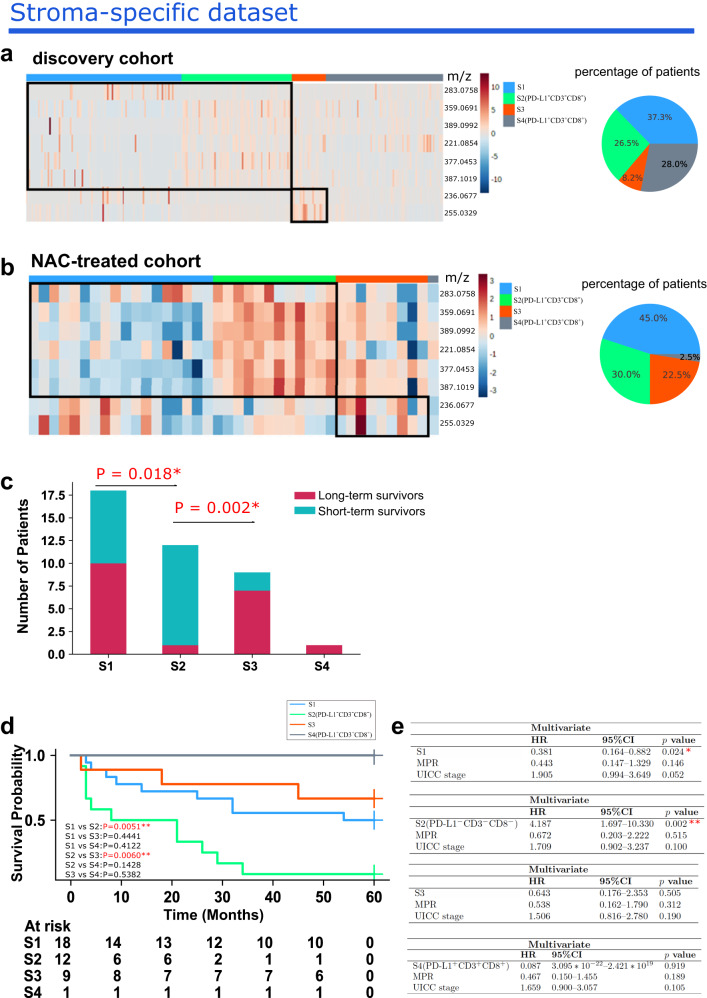
Association with chemotherapy response in the stroma-specific subtypes. Heatmap illustrating the abundance of metabolites shows stroma-specific subtype classification (**a**, left) in the discovery cohort and (**b**, left) NAC-treated cohort. The percentage of patients in the stroma-specific subtypes in the discovery cohort (**a**, right) and NAC-treated cohort (**b**, right). **c** Numbers of long-term survivors (chemotherapy-sensitive patients) and short-term survivors (chemotherapy-resistant patients) in the stroma-specific subtypes. Two-sided *p* value was calculated by Fisher’s exact test. **d** Survival comparison using log-rank test between overall and pairwise subtypes. **e** Multivariate Cox proportional hazard analysis for each of the stroma-specific subtypes, MPR as well as UICC stage. S1 and S2(PD-L1^-^CD3^-^CD8^-^) remain significant in multivariate analysis. * represents two-sided *p* < 0.05, ** represents two-sided *p* < 0.01, *** represents two-sided *p* < 0.001.

A previous study established a metabolomic classifier which comprises 100 metabolites to evaluate the response to chemotherapy in patients with non-small cell lung cancer^26^. This metabolomic classifier was established by applying spatial metabolomics and machine learning. The metabolomic classifier could stratify NSCLC patients into chemotherapy-sensitive and chemotherapy-resistant groups, and thus assess those patients’ response to chemotherapy. The metabolomic classifier of tumor and stroma were separately applied to associate chemotherapy responses in the established tumor- and stroma-specific subtypes. As shown in Fig. 5a, the stroma metabolomic classifier can distinguish the stroma-specific subtypes in our discovery cohort. In the NAC-treated cohort (*n* = 40), patients treated with chemotherapy were classified into the four stroma-specific subtypes (Fig. 5b). The proportion of chemotherapy-resistant patients was significantly higher in the S2(PD-L1^-^CD3^-^CD8^-^) subtype (92%) than in the S1 subtype (44%) (*p* = 0.018) and S3 subtype (22%) (*p* = 0.002) (Fig. 5c). In addition, chemotherapy-treated patients in the S2(PD-L1^-^CD3^-^CD8^-^) subtype also had a worse prognosis than patients in the S1 (*p* = 0.005) and S3 subtypes (*p* = 0.006) (Fig. 5d). Multivariate analysis shows that stroma-specific subtypes S1 and S2(PD-L1^-^CD3^-^CD8^-^) can serve as subtypes that are independently predictive of prognosis with regard to the major pathological response (MPR) and UICC classification system [S1: *p* = 0.024, HR = 0.381; S2(PD-L1^-^CD3^-^CD8^-^): *p* = 0.002, HR = 4.187] (Fig. 5e). No association of tumor-specific subtypes with chemotherapy response is found (Supplementary Fig. 3). Overall, these analyses demonstrate the correlation of these stroma-specific subtypes with survival and reveal their potential as biomarkers reflecting the response to chemotherapy.

## Discussion

**Fig. 6:**
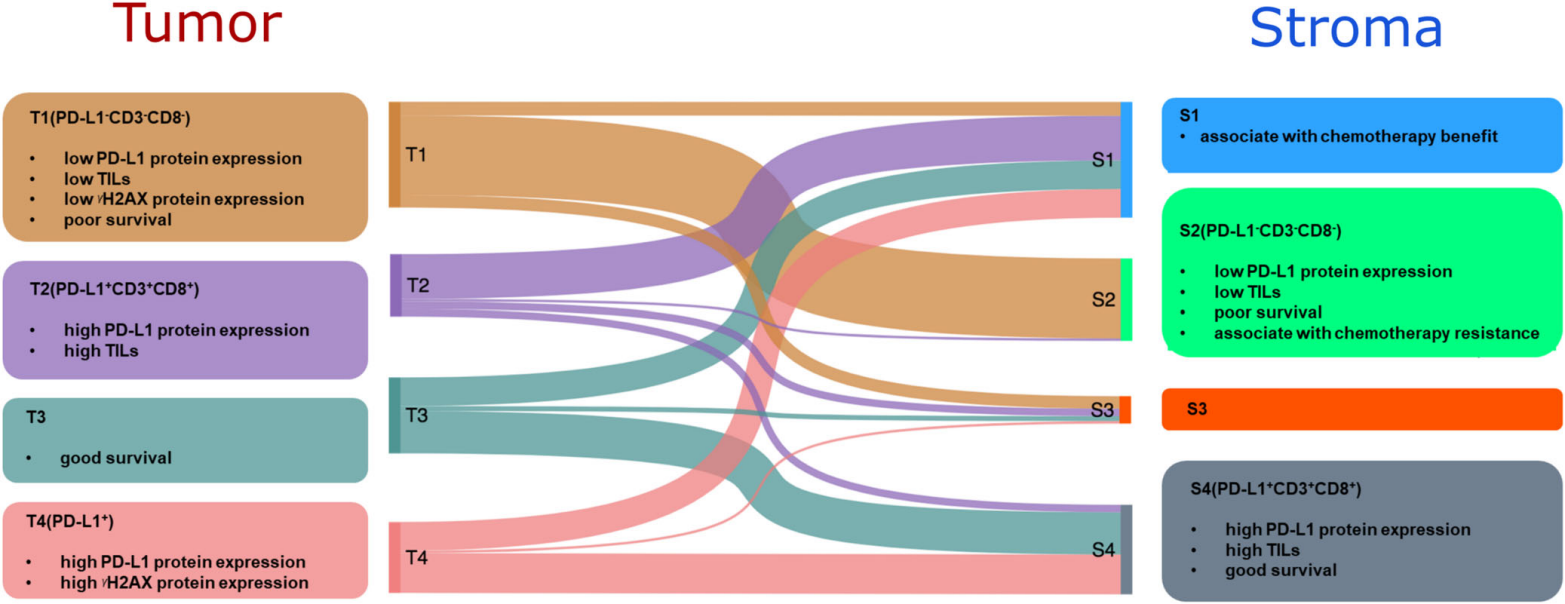
Summary of characteristics of the four tumor-specific and four stroma-specific LUSC patient subtypes. CD3: cluster of differentiation 3, CD8: cluster of differentiation 8, PD-L1: programmed death ligand 1, *γ*H2AX: DNA damage marker.

This study establishes metabolic subtypes in a large series of 330 patients with lung squamous cell carcinoma (LUSC). We define four distinct tumor-specific subtypes: T1(PD-L1^-^CD3^-^CD8^-^), T2(PD-L1^+^CD3^+^CD8^+^), T3, and T4(PD-L1^+^), and four stroma-specific subtypes: S1, S2(PD-L1^-^CD3^-^CD8^-^), S3, and S4(PD-L1^+^CD3^+^CD8^+^). The characteristics of these subtypes are summarized in Fig. 6. T1(PD-L1^-^CD3^-^CD8^-^) is characterized by low immune cell infiltration, low PD-L1 expression, low DNA damage (*γ*H2AX expression), and poor prognosis. By contrast, T2(PD-L1^+^CD3^+^CD8^+^) is characterized by high immune cell infiltration, high PD-L1 expression, and good prognosis; meanwhile, T3 has a favorable prognosis. Finally, T4(PD-L1^+^) is characterized by high PD-L1 expression. Stroma-specific subtypes are linked to immunological features and prognosis. An independent neoadjuvant chemotherapy-treated cohort (NAC-treated cohort) confirms that the S2(PD-L1^-^CD3^-^CD8^-^) subtype has an association with chemotherapy resistance. Taken together, our results suggest that distinct subtypes of LUSC as defined using metabolomics may show better responses to specific targeted therapies.

In recent years, molecular methods have been used for the classification of cancer into molecular subtypes^2–4,6–8,27^. Our subtype classification drew from these stratification approaches and supplemented them using tissue metabolomics to stratify LUSC patients. However, previous metabolomics stratification studies on patients with lung cancer focused largely on a mixture of tumor and stroma regions, analyzing few stromal regions from tumors and matching nonmalignant tissue. One study recently proposed distinctive stroma-based lung cancer subtypes by using single-cell RNA-sequencing^28^. In this study, we successfully separately performed the classification of tumor epithelial cells and stromal cells based on tissue-based spatial metabolomics. We found that the metabolic profiles of tumor and stroma tissues can be used to assign them to specific metabolic categories. Both tumor and stroma regions play important roles in the LUSC stratification, which could be confirmed by the tumor-specific subtype associations for several immune-related markers, including PD-L1, CD3, and CD8, being retained in the stroma-specific subtypes. However, stroma-specific subtypes were confirmed to be associated with chemotherapy response, while tumor metabolite signatures were not. This shows that tumor- and stroma-specific metabolite patterns from the same patient may convey different information, and the same patient cohort may have different subtype patterns in tumor- and stroma-specific regions. Thus, subtypes must be more precisely identified for individual tumor or stroma regions, rather than regions containing a mixture of tissues.

Several predictors of response to chemotherapy have been proposed in small cell lung cancer^29^. However, none yet provides a robust prediction of the benefit of chemotherapy in LUSC patients. There is thus an urgent need for a priori identification of responders to improve treatment outcomes. A metabolomic classifier was established in a previous study, and could assess the response to chemotherapy in patients with non-small cell lung cancer^26^. In the current study, LUSC patients from this recent study were used as an independent cohort and the identical metabolomic classifier was applied. Key metabolomic patterns distinguishing the LUSC stroma-specific subtypes, as first observed in the discovery cohort, were confirmed from the independent NAC-treated LUSC cohort. We successfully confirmed that our stroma-specific subtypes can further stratify patient responses to chemotherapy, with LUSC patients possessing S1 and S3 exhibiting better clinical responses to chemotherapy than S2(PD-L1^-^CD3^-^CD8^-^) patients. This evidence suggests that those assigned to the S1 and S3 subtypes are associated with a benefit from chemotherapy. In addition, the LUSC patients analyzed from the independent cohort could be classified into one of the four stroma-specific subtypes in the discovery cohort, raising the realistic possibility that prospective subtyping could be performed in a single trial, wherein patients are assigned to other treatment arms on the basis of their LUSC subtype [e.g., T2(PD-L1^+^CD3^+^CD8^+^) to PD-L1 immune checkpoint inhibitor].

To date, only immunotherapy has evolved into a successful therapeutic strategy for patients with LUSC^30,31^, but differences in patients’ responses to PD-1/PD-L1 inhibitors hinder its clinical application^32^. Effective prediction of the response to immunotherapy could dramatically enhance the proportion of patients who benefit while preventing overtreatment. T2(PD-L1^+^CD3^+^CD8^+^) captures several immunological features that are predictive of response to immunotherapy. The predictive biomarker for this immunotherapeutic class is PD-L1 overexpression^14,15^. Apart from this, the rate of tumor-infiltrating lymphocytes (TILs) is considered a potentially important predictive marker in a broad variety of tumor types^33–35^. In addition, pioneering studies in this field have confirmed a close correlation between TILs and PD1 overexpression in NSCLC^36–39^. Consequently, we expect T2(PD-L1^+^CD3^+^CD8^+^) to be susceptible to immune checkpoint inhibitors, such as PD-1/PD-L1 blockade, because of its positive association with PD-L1 expression, and CD3 and CD8^+^ T-cell infiltration. Besides the low expression of PD-L1, the T1(PD-L1^-^CD3^-^CD8^-^) subtype also shows low expression of CD3 and CD8. If confirmed in future studies, molecular classification might potentially be used to identify tumors of the T1(PD-L1^-^CD3^-^CD8^-^) subtype in order to select optimal treatment, particularly as these cases appear to represent an “immunologically ignorant” group unlikely to respond to immune checkpoint inhibitors.

To investigate the metabolites’ processes and events that play a role in the established tumor or stroma subtypes, we performed metabolic network analysis determining the correlations between endogenous metabolites. The metabolites that are correlated within each subtype comprise different classes of biomolecules, such as nucleotides and amino acids. These are involved in various pathways contributing to cancer cell growth and survival^40^. Cancer cells exhibit the deactivation of crucial DNA damage response signaling routes and have often undergone rewiring of their metabolism and energy production networks^41,42^. In addition, amino acids play a role in energy generation, maintaining cellular redox homeostasis and driving the synthesis of nucleic acids. Typically, alongside its association with the DNA damage-related protein *γ*H2AX, T1(PD-L1^-^CD3^-^CD8^-^) also 2’-demonstrates a dense cluster which strongly involves 2'-deoxycytidine diphosphate, cytidine diphosphate (CDP), uridine diphosphate (UDP), 2’-Deoxyinosine 5’-phosphate and cytidine in the metabolite network, which can be interpreted as an involvement in nucleotide metabolism occurring in response to DNA damage.

One major advantage of using FFPE TMAs in this study is the ability to directly detect and visualize metabolites, assigning them to specific tumor or stroma types in their native histological context. Compared to fresh frozen samples, FFPE TMAs offer superior morphological integrity^23^, enabling better tumor and stroma classification of metabolite content. A major limitation of using FFPE TMAs is the reduced or removed intensity of hydrophobic molecules. In a previous study, it was found that although metabolite peaks in the low mass range (*m*/*z* 50–400) were comparable to those in fresh frozen tissue, several peak intensities were decreased in the mass range above *m*/*z* 600^24^. The loss of hydrophobic molecules, for example, lipids, from the sample is a general limitation with FFPE patient samples due to the tissue embedding process and removal of paraffin wax via solvents, but there were classes of robust metabolites both chemically and spatially preserved in FFPE tissue specimens^23^. Moreover, many mass spectrometry studies, including those based on liquid- and gas-chromatography MS, have demonstrated that metabolites are reliably retained in FFPE tissue samples^43,44^. A recently published protocol for metabolomic and lipidomic profiling in FFPE kidney tissue by LC-MS with subsequent detection of selected lipid species by an independent in situ MS imaging approach demonstrates the complementary use of both techniques^45^.

In summary, our approach presented in this paper was successfully applied to reveal the ability of metabolomics to stratify LUSC patients. Such studies should aid in connecting metabolic profiles to clinical immunological features and in subsequently identifying therapeutic vulnerabilities and achieving effective, biomarker-based patient stratification.

## Methods

### Patients and tissue samples for the primary resected squamous cell lung carcinoma (LUSC) cohort

This study includes two retrospective single-center patient cohorts of primary resected and neoadjuvant chemotherapy-treated LUSC cases (Fig. 1). We analyzed 330 consecutive patients with primary resected LUSC^46^, diagnosed at the Institute of Tissue Medicine and Pathology, University of Bern, without previous or concomitant diagnosis of LUSC of other organs, to reliably exclude metastatic lung disease. The cohort of primary resected LUSC was resected and diagnosed during 2000–2013. The study was performed in accordance with the Declaration of Helsinki, and the local Ethics Committee of the Canton of Bern approved the study and waived the requirement for written informed consent (KEK 200/14). In this study, we used only tissue material from the archives of the Institute of Pathology which is left after the diagnostic process has been finalized. Due to the retrospective nature of the study and reusage of left-over or already collected material, and also due to the significant number of patients already deceased, the requirement for informed consent was waived by the local ethics committee. It was argued that contacting the relatives and the associated stress this would cause them would be disproportionate. Patients with documented refusal to participate in research, i.e., patients who refused that their tissue and data is used in retrospective research, had been excluded from the study. The cohort was assembled according to pathology files and validated according to clinical files. The histology of all cases was reassessed in accordance with current World Health Organization guidelines for the diagnosis of LUSC^47^. All tumors were restaged in accordance with the Union for International Cancer Control (UICC) 2017, 8th edition, tumor–node–metastasis (TNM) classification^48^. Disease-specific survival was defined as the duration from the date of diagnosis until death due to LUSC other than other causes. For patient characteristics, see Table 1. A tissue microarray was constructed from formalin-fixed, paraffin-embedded (FFPE) tissue blocks, as described previously^49^. Representative tissue blocks were selected for each tumor after reviewing all available slides per case (hematoxylin and eosin stained), and eight tumor cores were randomly selected from the block by placing digital annotations on the scanned slide. The eight cores were placed on tissue microarray blocks to exclude technical assessment bias.

**Table 1:** Summary of patient characteristics.

Characteristic	Numbers
Number of patients	330
Age [years]	
Median	69
Range	43–85
Sex	
Male	281 (85%)
Female	49 (15%)
pT stage	
T1	72 (22%)
T2	157 (48%)
T3	75 (22%)
T4	26 (8%)
pN stage	
N0	187 (57%)
N1	105 (32%)
N2	38 (11%)
M	
M0	321 (97%)
M1	9 (3%)
UICC stage	
I	98 (30%)
II	113 (34%)
III	110 (33%)
IV	9 (3%)
Primary resection status	
R0	287 (87%)
R1	40 (12%)
R2	3 (1%)
Grade	
G1	6 (2%)
G2	163 (49%)
G3	161 (49%)

M: distant metastases (M0: absent; M1: present).

### Patients and tissue samples for the independent neoadjuvant chemotherapy-treated cohort

The NAC-treated cohort comprises 40 cases^26^ diagnosed at the Institute of Pathology of the University of Bern between 2000 and 2016. All eligible patients had a pathology-confirmed diagnosis. The NAC-treated cohort was separated into long-term (*n* = 19) and short-term survivors (*n* = 21) according to median overall survival^26^. The cohort included consecutive patients who received at least one cycle of platinum-based chemotherapy prior to resection (Supplementary Table 1). The study was approved by the Cantonal Ethics Commission of the Canton of Bern (KEK 2017-00830), which waived the requirement for a written informed consent from patients. Due to the retrospective nature of the study and reusage of left-over or already collected material, and also due to the significant number of patients already deceased, the requirement for informed consent was waived by the local ethics committee. It was argued that contacting the relatives and the associated stress this would cause them would be disproportionate. Patients with documented refusal to participate in research, i.e., patients who refused that their tissue and data is used in retrospective research, had been excluded from the study. A tissue microarray was constructed from FFPE tissue blocks. The NAC-treated cohort was integrated for an independent study for evaluating the response to chemotherapy of the metabolic subtypes.

### High-mass-resolution MALDI-Fourier transform ion cyclotron resonance (FT-ICR) IMS

Data for spatial metabolomics of the primary resected LUSC cohort and NAC-treated cohort were obtained from previous studies^26,46^. High-mass-resolution MALDI FT-ICR IMS was performed as previously described^23^. In brief, FFPE sections (4 μm) were mounted onto indium–tin–oxide (ITO)-coated glass slides (Bruker Daltonik). The air-dried tissue sections were spray-coated with 10 mg/mL 9-aminoacridine hydrochloride monohydrate matrix (Sigma-Aldrich) in methanol (70%) using the SunCollect sprayer (Sunchrom). Spray-coating of the matrix was conducted in eight passes, utilizing a line distance of 2 mm and a spray velocity of 900 mm/min.

Metabolites were detected in negative-ion mode on a 7 T Solarix XR FT-ICR mass spectrometer (Bruker Daltonik) equipped with a dual electrospray ionization MALDI (ESI-MALDI) source and a SmartBeam-II Nd:YAG (355 nm) laser. The SCiLS Lab software 2020b was used to export the selected peaks of the mass spectra as processed and root mean square-normalized imzML files. Peak annotations were based on accurate mass matching with the Human Metabolome Database (HMDB) (https://hmdb.ca/) and Kyoto Encyclopedia of Genes and Genomes (KEGG) database (https://www.genome.jp/kegg/).

### Immunophenotype-guided IMS and data processing

The SPACiAL workflow was used as previously described^25^ to automatically annotate tumor and stroma regions in LUSC tissues in the primary resected cohort and NAC-treated cohort (Supplementary Fig. 4). SPACiAL is a computational multimodal workflow that includes a series of image and MALDI data processing steps to combine molecular imaging data with multiplex immunofluorescence. First, after MALDI–IMS analysis, the 9-aminoacridine matrix was removed from tissue sections, followed by immunofluorescence staining. Double staining of the TMA was performed using the epithelial marker pan-cytokeratin [monoclonal mouse pan-cytokeratin plus (AE1/AE3þ8/18), 1:75, catalog no. CM162; Biocare Medical] and vimentin (Abcam, clone ab92547, 1:500). Second, single-channel images of pan-cytokeratin and vimentin were used to annotate and separate tumor and stroma using fluorescence imaging. Regions positive for pan-cytokeratin were defined as tumor. Regions negative for pan-cytokeratin but positive for vimentin were defined as stroma; third, the digitized and co-registered fluorescence images were scaled to match the exact MALDI resolution and converted into numerical matrices comprised of values corresponding to the lightness values for each pixel; fourth, objective tissue annotations were assigned based on semantics and function. The annotatable patient cases formed the basis of our calculations. The entire workflow is applied to the same tissue section, allowing for the automatic integration of morphological and spatial metabolomics data for thousands of molecules.

### Immunohistochemistry (IHC)

IHC staining for cluster of differentiation 3 (CD3), cluster of differentiation 8 (CD8), and programmed death ligand 1 (PD-L1) was performed as previously described^49^ on consecutive sections. In brief, an automated immunostainer (Bond III, Leica Bio-systems) with anti-CD3 (Abcam Cambridge; clone SP7, 1:400, RRID: AB_443425), anti-CD8 (Dako, clone C8/144B, 1:100, RRID: AB_2075537), and anti-PD-L1 (Cell Signaling Technology, clone E1L3N, 1:400, RRID: AB_2687655) was used. CD3 and CD8 expression was determined using image analysis (Aperio Image Scope) and adjusted for core completeness. PD-L1 expression was assessed by a pathologist (S. Berezowska) as the proportion of positive tumor cells.

### Immunofluorescence analysis of ***γ***H2AX

Immunofluorescence analysis of *γ*H2AX expression was achieved using primary antibodies against pH2A.X (Cell Signaling Technology; catalog no. 2577, 1:400, RRID: AB_2118010) and pan-cytokeratin [monoclonal mouse pan-cytokeratin plus (AE1/AE3þ8/18), 1:75, catalog no. CM162; Biocare Medical] on consecutive sections. Slides were digitized at ×20 objective magnification using an Axio Scan.Z1 (Zeiss). Quantification was performed by digital image analysis in Definiens Developer XD2, following a previously published procedure^50^. The quantified parameter was the proportion of *γ*H2AX- and pan-cytokeratin-positive cells to the total number of pan-cytokeratin-positive cells.

### Consensus clustering

Consensus clustering was conducted using the ‘ConcensusClusterPlus’ package in R using HMDB-annotated metabolites to explore LUSC subtypes based on the patient sample matrix. The consensus matrix was used to check cluster co-occurrence, find intrinsic groupings over variation in different numbers of clusters, and use hierarchical clustering on the distance matrix. We used a prespecified subsampling parameter of 80% with 1000 iterations and assigned the number of potential clusters (K) to range from 2 to 10 in order to avoid producing an excessive number of clusters that would not be clinically useful. The matrix was arranged so that samples belonging to the same cluster were adjacent to each other.

### Correlation network analysis and quantitative pathway analysis

Correlation networks were created using Cytoscape (v. 3.8.0). All networks were visualized using the absolute value of the correlation coefficient calculated by Spearman’s rank-order correlation. Metabolites with at least one significant correlation are shown (*p* < 0.001). Quantitative pathway analysis was performed via the KEGG database using the MetaboAnalyst online tool (www.metaboanalyst.ca) based on the correlated metabolites.

### Statistical analysis

All statistical tests were conducted using Python or R. Correlations were calculated using Spearman’s rank-order correlation. The significance of differences in clinicopathological characteristics among tumor- and stroma-specific subtypes was evaluated by chi-squared test or Fisher’s exact test. To determine the intensity of differences of representative metabolites, Kruskal–Wallis test and post hoc Dunn’s multiple comparison test were used in conjunction with Benjamini–Hochberg correction. Further comparisons to identify the statistical significance of differences in patient survival were performed using the Kaplan–Meier curve and the log-rank test. Multivariate survival analysis was performed using Cox proportional hazard regression model. A two-sided *p* value of <0.05 was considered statistically significant.

### Reporting summary

Further information on research design is available in the Nature Research Reporting Summary linked to this article.

### Supplementary information

Supplementary Information

Reporting Summary

## Data Availability

The datasets generated during and/or analyzed during the current study are available from the corresponding author on reasonable request. Full microscopy image datasets are deposited in BioStudies under the accession number S-BIAD814 at https://www.ebi.ac.uk/biostudies/bioimages/studies/S-BIAD814.
