# Supplementary material for: Spatial metabolomics identifies distinct tumor-specific and stroma-specific subtypes in patients with lung squamous cell carcinoma
Source: NPJ Precis Oncol. 2023 Nov 2;7:114. doi: 10.1038/s41698-023-00434-4 (PMC10622419; doi:10.1038/s41698-023-00434-4)
Supplement: Supplementary file 1 — Supplementary Information [file 41698_2023_434_MOESM1_ESM.pdf]

SUPPLEMENTARY INFORMATION

Supplementary Table 1: Summary of patient characteristics in the NAC-treated cohort. MPR was defined as ⩽10% residual tumor cells. Samples with insufficient data to make a conclusion are indicated as "NA."

Characteristic	Long-term survivors	Short-term survivors
**Number of patients**	19	21
**Sex**		
Male	14	17
Female	5	4
**Age [years]**		
Median	60	62
Range	46–73	46–77
**MPR**		
Present	11	4
Absent	8	17
**UICC stage**		
0	2	1
1	7	0
2	5	6
3	5	12
4	0	1
NA	0	1

Supplementary Table 2: Spearman's correlation of molecular features with tumor-specific subtypes.

Subtype	Protein	Coefficient	p value
T1(PD-L1$^-$CD3$^-$CD8$^-$)	CD3	−0.1736	0.0002
T1(PD-L1$^-$CD3$^-$CD8$^-$)	CD8	−0.2181	0.0001
T1(PD-L1$^-$CD3$^-$CD8$^-$)	PD-L1	−0.1826	0.0012
T1(PD-L1$^-$CD3$^-$CD8$^-$)	γH2AX	−0.1804	0.0016
T2(PD-L1$^+$CD3$^+$CD8$^+$)	CD3	0.1518	0.0071
T2(PD-L1$^+$CD3$^+$CD8$^+$)	CD8	0.1502	0.0080
T2(PD-L1$^+$CD3$^+$CD8$^+$)	PD-L1	0.1169	0.0390
T2(PD-L1$^+$CD3$^+$CD8$^+$)	γH2AX	0.0966	0.0933
T3	CD3	0.0407	0.4732
T3	CD8	0.0567	0.3178
T3	PD-L1	0.0233	0.6809
T3	γH2AX	−0.0189	0.7428
T4(PD-L1$^+$)	CD3	−0.0006	0.9919
T4(PD-L1$^+$)	CD8	0.0769	0.1755
T4(PD-L1$^+$)	PD-L1	0.2402	0.0004
T4(PD-L1$^+$)	γH2AX	0.1223	0.0333

Supplementary Table 3: Spearman's correlation of molecular features with stroma-specific subtypes.

Subtype	Protein	Coefficient	p value
S1	CD3	−0.0844	0.1697
S1	CD8	−0.0503	0.4126
S1	PD-L1	−0.0075	0.9033
S1	γH2AX	−0.0207	0.7397
S2(PD-L1$^-$CD3$^-$CD8$^-$)	CD3	−0.1755	0.0041
S2(PD-L1$^-$CD3$^-$CD8$^-$)	CD8	−0.2070	0.0007
S2(PD-L1$^-$CD3$^-$CD8$^-$)	PD-L1	−0.1681	0.0058
S2(PD-L1$^-$CD3$^-$CD8$^-$)	γH2AX	−0.0563	0.3660
S3	CD3	0.1198	0.0510
S3	CD8	0.1072	0.0804
S3	PD-L1	0.0073	0.9050
S3	γH2AX	−0.0308	0.6214
S4(PD-L1$^+$CD3$^+$CD8$^+$)	CD3	0.1897	0.0019
S4(PD-L1$^+$CD3$^+$CD8$^+$)	CD8	0.1912	0.0017
S4(PD-L1$^+$CD3$^+$CD8$^+$)	PD-L1	0.1688	0.0056
S4(PD-L1$^+$CD3$^+$CD8$^+$)	γH2AX	0.0956	0.1242

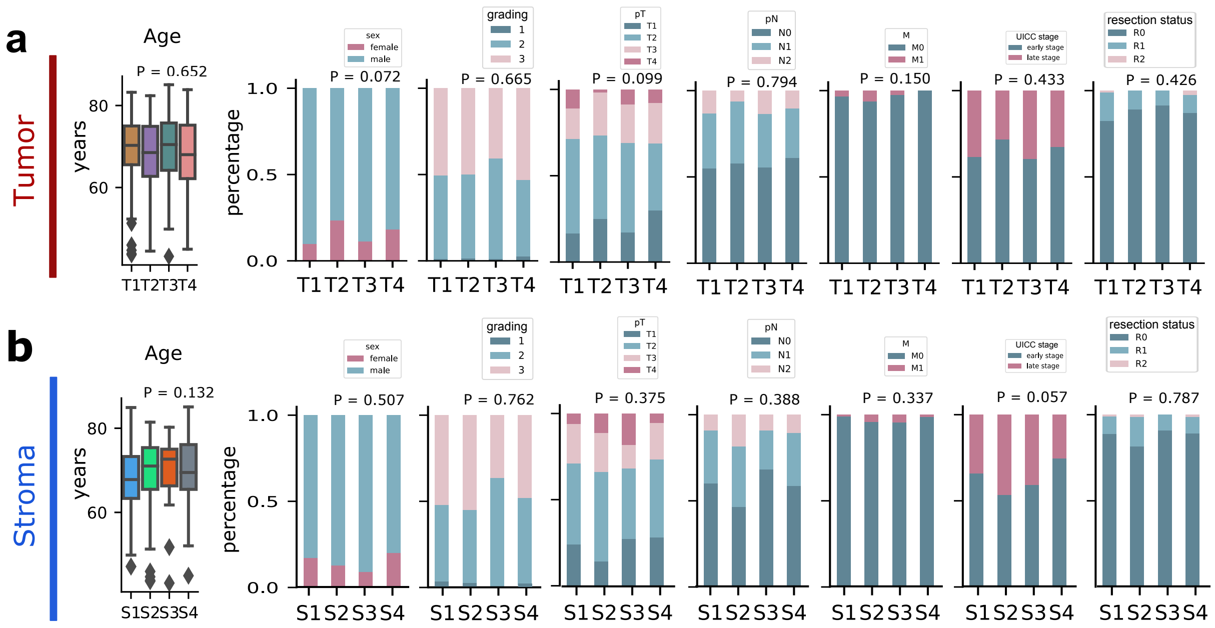

Supplementary Figure 1: Clinicopathological characteristics of patient subtypes. Differences in clinicopathological characteristics among (a) tumor- and (b) stroma-specific subtypes were analyzed by chi-squared test or Fisher's exact test. The variable of age was tested by Kruskal–Wallis test. Each box plot displays the interquartile range (IQR), with the lower boundary representing the 25th percentile and the upper boundary representing the 75th percentile. The line within the box displays the median, and the whiskers extend to $\pm 1.5 \times$ IQR.

Stroma-specific dataset

S1

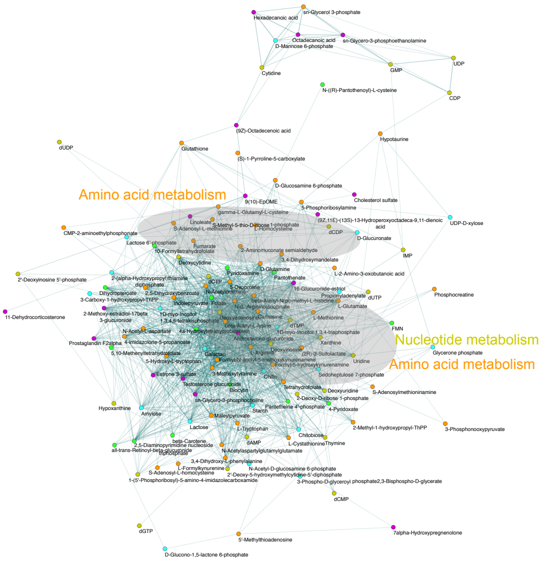

S3

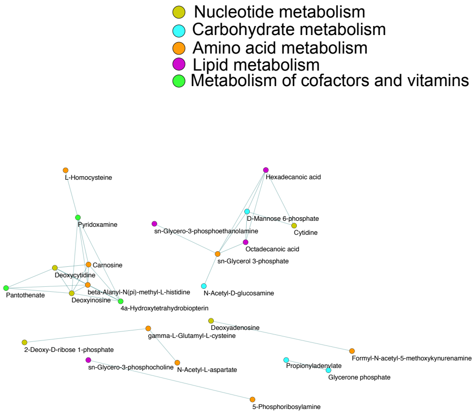

enriched pathways of S1

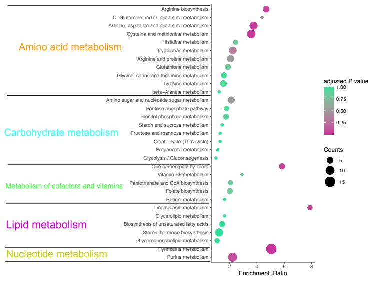

enriched pathways of S3

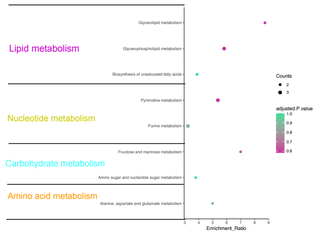

S2(PD-L1$^-$CD3$^-$CD8$^-$)

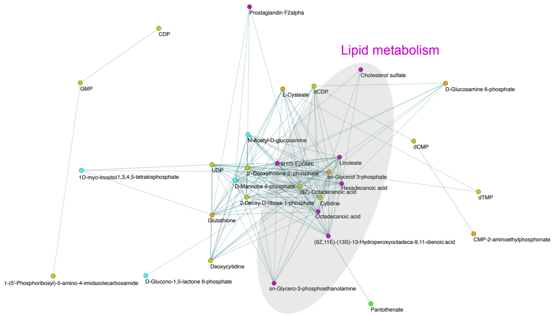

S4(PD-L1$^+$CD3$^+$CD8$^+$)

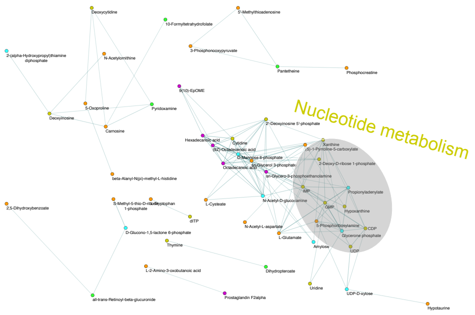

enriched pathways of S2(PD-L1$^-$CD3$^-$CD8$^-$)

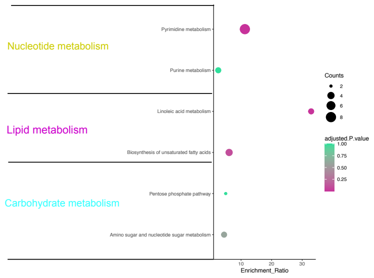

enriched pathways of S4(PD-L1$^+$CD3$^+$CD8$^+$)

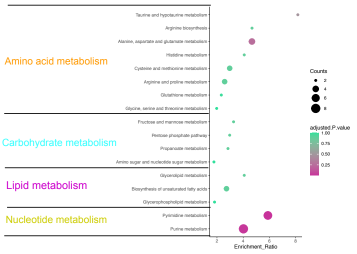

Supplementary Figure 2: Metabolite characteristics of stroma-specific subtypes. (top) Correlation networks of endogenous metabolites and pathway analyses within each of the four stroma-specific subtypes. Correlations between metabolites were calculated and filtered (adjusted two-sided $p < 0.001$). Edges represent positive (green) and negative (pink) correlations between metabolites. Node color in the network indicates metabolic pathways. (bottom) Quantitative enrichment pathway analysis within each of the four stroma-specific subtypes. Pathways enriched in each of the stroma-specific subtypes are represented by scatter plots. The x-axis indicates the pathway enrichment ratio, and the y-axis indicates the pathway term. Dot color indicates the adjusted p value. Dot size indicates the counts of metabolites.

Tumor-specific dataset

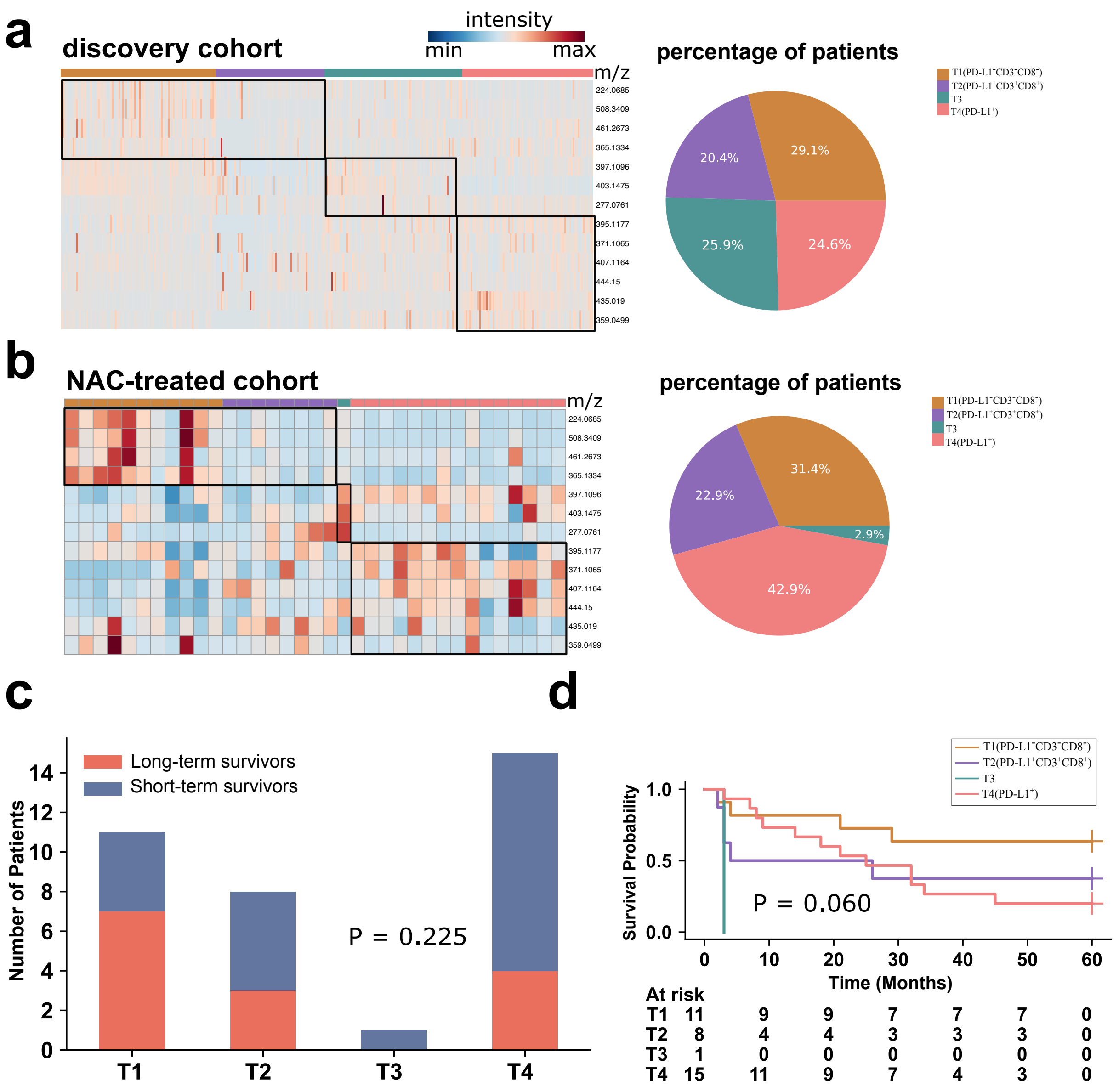

Supplementary Figure 3: Association with chemotherapy response in the tumor-specific subtypes. Heatmap illustrating the abundance of metabolites shows tumor-specific subtype classification (a, left) in the discovery cohort and (b, left) NAC-treated cohort. The proportions of patients in the tumor-specific subtypes in the discovery cohort (a, right) and NAC-treated cohort (b, right). (c) Numbers of long-term survivors (chemotherapy-sensitive patients) and short-term survivors (chemotherapy-resistant patients) in the tumor-specific subtypes. Two-sided p value was calculated by Fisher's exact test. (d) Survival comparison among patient subtypes using log-rank test.

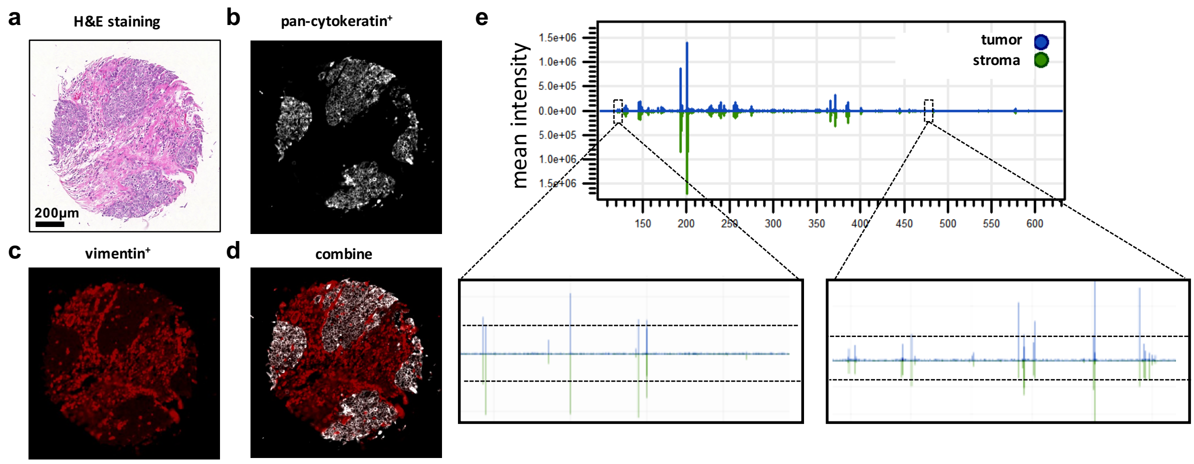

Supplementary Figure 4: Multiplex immunophenotype-guided imaging mass spectrometry for metabolomic analysis of tumor and stroma regions. (a) Hematoxylin and eosin staining of a representative tissue core. (b, c) Single channel images of the tumor marker (pan-cytokeratin) and the stromal marker (vimentin) used to annotate and separate tumor and stroma cells by fluorescence imaging. (d) Representative double-channel images of the tissue core stained with pan-cytokeratin (white) and vimentin (red). (e) Juxtaposition of the mean spectrum from tumor (white) and stroma (red) regions in (d), represented as mean intensity. The mean spectra of tumor and stroma region indicates a different metabolic profile.
