# Supplementary material for: Spatial metabolomics identifies distinct tumor-specific and stroma-specific subtypes in patients with lung squamous cell carcinoma
Source: NPJ Precis Oncol. 2023 Nov 2;7:114. doi: 10.1038/s41698-023-00434-4 (PMC10622419; doi:10.1038/s41698-023-00434-4)
Supplement: Supplementary file 2 — Reporting Summary [file 41698_2023_434_MOESM2_ESM.pdf]

nature portfolio

Corresponding author(s): Axel Walch

Last updated by author(s): May 23, 2023

Reporting Summary

Nature Portfolio wishes to improve the reproducibility of the work that we publish. This form provides structure for consistency and transparency in reporting. For further information on Nature Portfolio policies, see our Editorial Policies and the Editorial Policy Checklist.

Statistics

For all statistical analyses, confirm that the following items are present in the figure legend, table legend, main text, or Methods section.

n/a	Confirmed	
☐	☒	The exact sample size (*n*) for each experimental group/condition, given as a discrete number and unit of measurement
☐	☒	A statement on whether measurements were taken from distinct samples or whether the same sample was measured repeatedly
☐	☒	The statistical test(s) used AND whether they are one- or two-sided<br>*Only common tests should be described solely by name; describe more complex techniques in the Methods section.*
☐	☒	A description of all covariates tested
☐	☒	A description of any assumptions or corrections, such as tests of normality and adjustment for multiple comparisons
☐	☒	A full description of the statistical parameters including central tendency (e.g. means) or other basic estimates (e.g. regression coefficient) AND variation (e.g. standard deviation) or associated estimates of uncertainty (e.g. confidence intervals)
☐	☒	For null hypothesis testing, the test statistic (e.g. *F*, *t*, *r*) with confidence intervals, effect sizes, degrees of freedom and *P* value noted<br>*Give P values as exact values whenever suitable.*
☒	☐	For Bayesian analysis, information on the choice of priors and Markov chain Monte Carlo settings
☒	☐	For hierarchical and complex designs, identification of the appropriate level for tests and full reporting of outcomes
☐	☒	Estimates of effect sizes (e.g. Cohen's *d*, Pearson's *r*), indicating how they were calculated

Our web collection on statistics for biologists contains articles on many of the points above.

Software and code

Policy information about availability of computer code

Data collection	SCiLSLab 2020b; Fleximaging 5.0
Data analysis	MetaboAnalyst 5.0; Cytoscape 3.8.0; HMDB 4.0; KEGG; R; Python

For manuscripts utilizing custom algorithms or software that are central to the research but not yet described in published literature, software must be made available to editors and reviewers. We strongly encourage code deposition in a community repository (e.g. GitHub). See the Nature Portfolio guidelines for submitting code & software for further information.

Data

Policy information about availability of data

All manuscripts must include a data availability statement. This statement should provide the following information, where applicable:

- Accession codes, unique identifiers, or web links for publicly available datasets
- A description of any restrictions on data availability
- For clinical datasets or third party data, please ensure that the statement adheres to our policy

The datasets generated during and/or analysed during the study are available from the corresponding author on reasonable request.

Research involving human participants, their data, or biological material

Policy information about studies with human participants or human data. See also policy information about sex, gender (identity/presentation), and sexual orientation and race, ethnicity and racism.

Reporting on sex and gender	This study includes two patient cohorts. The overall numbers of patients is 330 for the primary resected cohort in this study. The term sex was used as biological attribute with 281 male patients and 49 female patients. Sex was used to test whether it differed tumor-specific and stroma-specific subtypes. The neoadjuvant chemotherapy-treated cohort with 40 patients does not report sex or gender in this study.
Reporting on race, ethnicity, or other socially relevant groupings	No socially constructed or socially relevant categorization variables were used in the manuscript.
Population characteristics	Patient characteristics are given in Table 1 and Supplementary table 1.
Recruitment	This study includes two patient cohorts of primary resected and neoadjuvant chemotherapy-treated squamous cell lung carcinoma (LUSC) cases. All eligible patients had a pathology-confirmed diagnosis. All patients were diagnosed at the Institute of Pathology of the University of Bern. We did not preselect patients in terms of age, gender or comorbidities.
Ethics oversight	The study was performed in accordance with the Declaration of Helsinki, and the local Ethics Committee of the Canton of Bern approved the study and waived the requirement for written informed consent.

Note that full information on the approval of the study protocol must also be provided in the manuscript.

Field-specific reporting

Please select the one below that is the best fit for your research. If you are not sure, read the appropriate sections before making your selection.

☒ Life sciences ☐ Behavioural & social sciences ☐ Ecological, evolutionary & environmental sciences

For a reference copy of the document with all sections, see nature.com/documents/nr-reporting-summary-flat.pdf

Life sciences study design

All studies must disclose on these points even when the disclosure is negative.

Sample size	This study includes the number of patients 330 in the primary resected cohort and 40 in the neoadjuvant chemotherapy-treated cohort.
Data exclusions	No data were excluded.
Replication	During the tissue microarrays construction process, representative tissue blocks were selected for each tumor after reviewing all available slides per case, and eight tumor cores were randomly selected from the tumor tissue block by placing digital annotations on the scanned slide. The eight cores were placed on tissue microarray blocks to exclude technical assessment bias. All attempts at replication was successful.
Randomization	The tissue microarrays were randomized for matrix-assisted laser desorption ionization (MALDI) mass spectrometry imaging measurement .
Blinding	Blinding is not relevant since this study did not separate patients into experimental groups.

Reporting for specific materials, systems and methods

We require information from authors about some types of materials, experimental systems and methods used in many studies. Here, indicate whether each material, system or method listed is relevant to your study. If you are not sure if a list item applies to your research, read the appropriate section before selecting a response.

Materials & experimental systems

n/a	Involved in the study	
☐	☒	Antibodies
☒	☐	Eukaryotic cell lines
☒	☐	Palaeontology and archaeology
☒	☐	Animals and other organisms
☒	☐	Clinical data
☒	☐	Dual use research of concern
☒	☐	Plants

Methods

n/a	Involved in the study	
☒	☐	ChIP-seq
☒	☐	Flow cytometry
☒	☐	MRI-based neuroimaging

Antibodies

Antibodies used	anti-CD3, Abcam Cambridge, rabbit monoclonal, Cat# ab16669; anti-CD8, Agilent, mouse monoclonal, Cat# M7103; anti-PD-L1, Cell Signaling Technology, rabbit monoclonal, Cat# 13684; Cell Signaling Technology, pH2A.X, rabbit polyclonal, Cat# 2577.
Validation	Before usage for all antibodies, a system control was completed. An adequate negative and positive control was carried along for each staining procedure. Immunohistochemistry for anti-CD3 was tested by the company. Anti-CD3 was validated by the Abcam company for western blot analysis(WB) and immunohistochemistry (IHC) and flow cytometry. Anti-CD8 was validated by the Agilent company for IHC. Anti-PD-L1 was validated by the CST company for WB, Immunoprecipitation, IHC and flow cytometry. pH2A.X was validated by the CST company for WB and immunofluoresence and flow cytometry.
